# Knock-in human GDF5 proregion L373R mutation as a mouse model for proximal symphalangism

**DOI:** 10.18632/oncotarget.23047

**Published:** 2017-12-08

**Authors:** Xinxin Zhang, Xuesha Xing, Xing Liu, Yu Hu, Shengqiang Qu, Heyi Wang, Yang Luo

**Affiliations:** ^1^ The Research Center for Medical Genomics, Key Laboratory of Cell Biology, Ministry of Public Health, Key Laboratory of Medical Cell Biology, Ministry of Education, College of Basic Medical Science, China Medical University, Shenyang, Liaoning, China

**Keywords:** GDF5, SYM1, proregion, knock-in, gain-of-function

## Abstract

Proximal symphalangism (SYM1) is an autosomal dominant disorder, mainly characterized by bony fusions of the proximal phalanges of the hands and feet. *GDF5* and *NOG* were identified to be responsible for SYM1. We have previously reported on a p.Leu373Arg mutation in the GDF5 proregion present in a Chinese family with SYM1. Here, we investigated the effects of the GDF-L373R mutation. The variant caused proteolysis efficiency of GDF5 increased in ATDC5 cells. The variant also caused upregulation of SMAD1/5/8 phosphorylation and increased expression of target genes *SMURF1*, along with *COL2A1* and *SOX9* which are factors associated with chondrosis. Furthermore, we developed a human-relevant SYM1 mouse model by making a *Gdf5*^*L367R*^ (the orthologous position for L373R in humans) knock-in mouse. *Gdf5*^*L367R/+*^ and *Gdf5*^*L367R/L367R*^ mice displayed stiffness and adhesions across the proximal phalanx joint which were in complete accord with SYM1. It was also confirmed the joint formation and development was abnormal in *Gdf5*^*L367R/+*^ and *Gdf5*^*L367R/L367R*^ mice, including the failure to develop the primary ossification center and be hypertrophic chondrocytes during embryonic development. This knock-in mouse model offers a tool for assessing the pathogenesis of SYM1 and the function of the GDF5 proregion.

## INTRODUCTION

Proximal symphalangism (SYM1, OMIM 185800), also known as “straight hands”, is a dominant hereditary developmental disorder. The disease manifests as fusions of the proximal interphalangeal joints, typically on the third to fifth digits of the hand, and also affects the thumb, making it impossible to make a fist. The bony fusions occur on metacarpophalangeal joints as well as on the auditory ossicles, leading to conductive hearing loss in some affected individuals [1, 2]. The disease results from mutations of the *growth differentiation factor 5 (GDF5)* or *NOG* genes [1].

The *GDF5* gene, also called *CDMP1*, is located on 20q11.22. GDF5 plays important roles during early and later limb development, as well as in various musculoskeletal physiological processes [3]. It also contributes to the structural and functional maintenance of the intervertebral disc [4]. GDF5 belongs to the transforming growth factor beta (TGFβ) superfamily. It has a similar conserved structure and shares the same signal transduction mechanism with other family members in the TGFβ superfamily [5]. GDF5 contains an N terminal signal peptide domain, a large proregion comprised of 354 residues, and a 120 amino acid mature peptide at the carboxyl terminus [6].

Mutations in *GDF5* are associated with various autosomal recessive and dominant syndromes related to skeletal abnormalities, such as Brachydactyly C (BDC, OMIM, 113100), Acromesomelic chondrodysplasia Grebe type (AMDG, OMIM, 200700), Du Pan syndrome (DPS, OMIM, 228900), Symphalangism proximal 1B (SYM1B, OMIM, 615298), Multiple synostoses syndrome 2 (SYNS2, OMIM, 610017) and Brachydactyly A2 (BDA2, OMIM, 112600) [7–10]. Until now, only five mutations in GDF5 (R438L, E491K, N445K/T, and L373R) have been identified that are related with SYM1, and of those only L373R is located in the proregion, which was previously reported on by our group [11–15].

Here, we developed a human-relevant SYM1 mouse model by making a *Gdf5*^*L367R*^ (the orthologous position as L373R in human) knock-in mice. The clinical phenotype of proximal symphalangism and similar histology of skeletal abnormalities were observed in the mutant mice. This offers a valuable tool for assessing the pathogenesis of SYM1 and characterizing the abnormal function of mutant GDF5.

## RESULTS

### Functional analyses of the variant of GDF5^L373R^

**Figure 1:**
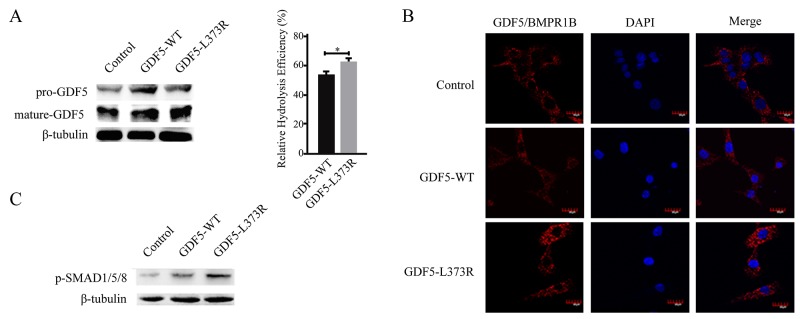
Functional analyses of the GDF5-L373R mutation **(A)** Expression of pre-GDF5 and mature-GDF5 after transfection with wildtype or mutant GDF5 vector lacking the signal peptide (left).The relative hydrolysis efficiency of GDF5 was represented by the percentage of mature GDF5 protein compared to total GDF5 protein (mature-GDF5/ pre-GDF5 + mature-GDF5) (right). **(B)** Confocal images showing Duolink fluorescence alone (GDF5/BMPR1B interaction, red, center column), DAPI (blue, center column), and merged (right column). **(C)** The phosphorylation of SMAD1/5/8. (^*^P <0.05).

We first investigated the proteolysis efficiency of the mutant protein because of the special location of the GDF5^L373R^ mutation. Expression vectors with wildtype or L373R mutant GDF5, both lacking signal peptides, were transfected into ATDC5 cells. The percentage of mature GDF5 protein compared to total GDF5 protein (the summation of mature GDF5 and proprotein GDF5 protein) was assessed to indicate the relative hydrolysis efficiency of the GDF5 protein. The results of the Western Blotting suggested that the GDF5^L373R^ mutation increased the proteolysis efficiency of the protein (Figure 1A).

Next, we detected the interaction between GDF5 and its high affinity receptor, BMPR1B, using a Duolink in situ interaction assay. Increased Duolink staining in GDF5^L373R^ than GDF5^WT^ cells indicated that the L373R mutation might increase the interaction between GDF5 and BMPR1B (Figure 1B). The SMAD signaling cascade is known to be activated upon binding of GDF5 to its receptors, and SMAD1/5/8 phosphorylation was seen at a higher level in cells infected with GDF5^L373R^ than with GDF5^WT^ (Figure 1C).

**Figure 2:**
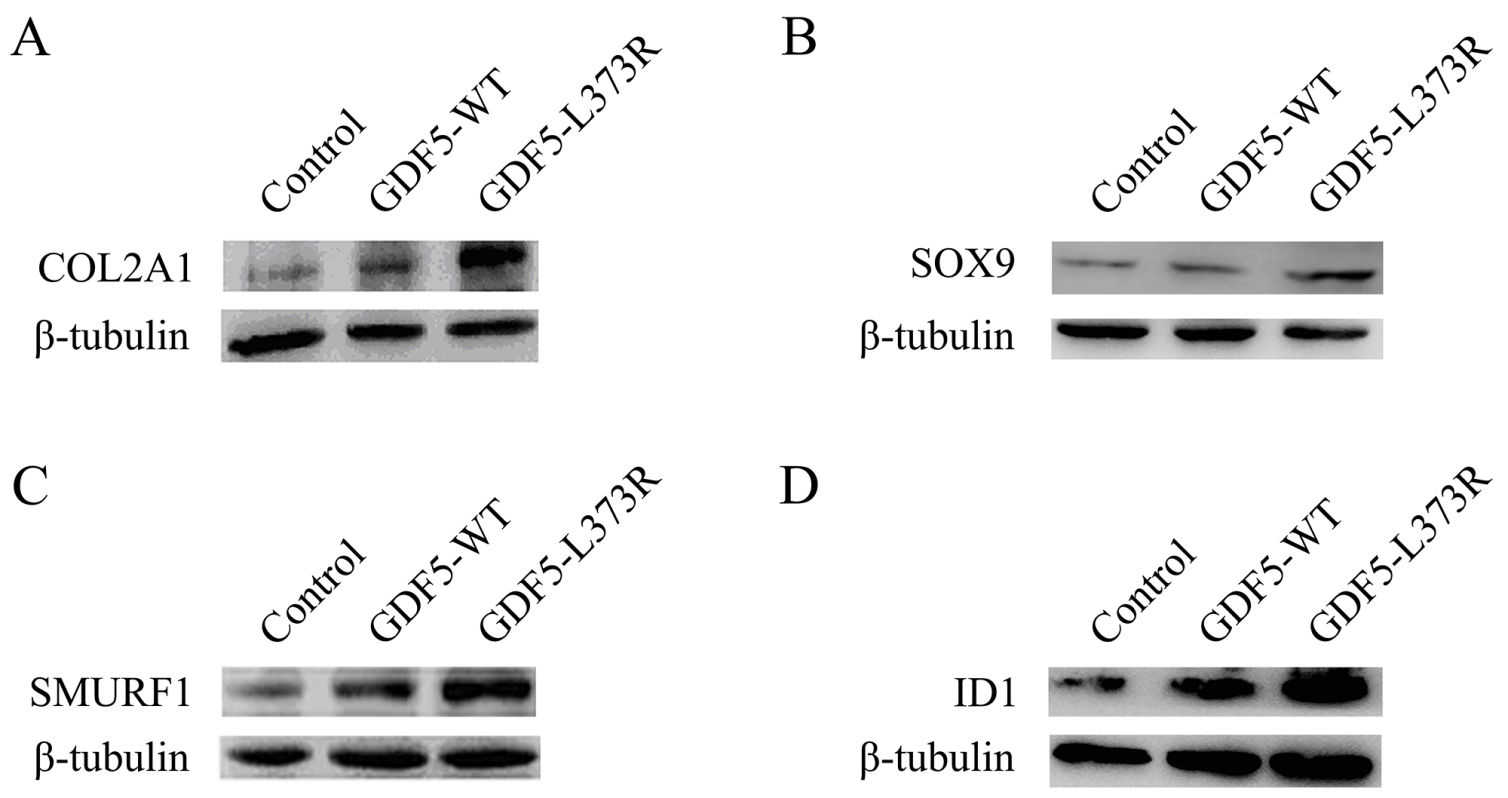
Effects of GDF5-L373R mutation in factors associated with chondrosis Expression of COL2A1 **(A)**, SOX9 **(B)**, SMURF1 **(C)** and ID 1 **(D)** in ATDC5 cells infected with control, GDF5-WT, or GDF5-L373R virus.

After that we explored the alterations of related factors affected by GDF5^L373R^ during chondrosis and skeletal development. Collagen type II alpha 1 chain (COL2A1), a cartilage-specific matrix protein, and SRY-box 9 (SOX9), a high-mobility-group transcription factor required for chondrogenic differentiation of mesenchyme and chondrocyte maturation [16], were seen to be upregulated in cells infected with the GDF5^L373R^ virus (Figure 2A, 2B). The expression of another important regulatory factor for bone formation, Smad ubiquitination regulatory factor-1 (SMURF1), was also increased in cells infected with the GDF5^L373R^ virus (Figure 2C). In contrast, infection with the GDF5^L373R^ virus resulted a reduction of inhibitor of differentiation 1 (ID1), which negatively regulates the expression of COL2A1 and SOX9 (Figure 2D).

### Generation of *GDF5*^*SYM1*^ mice

**Figure 3:**
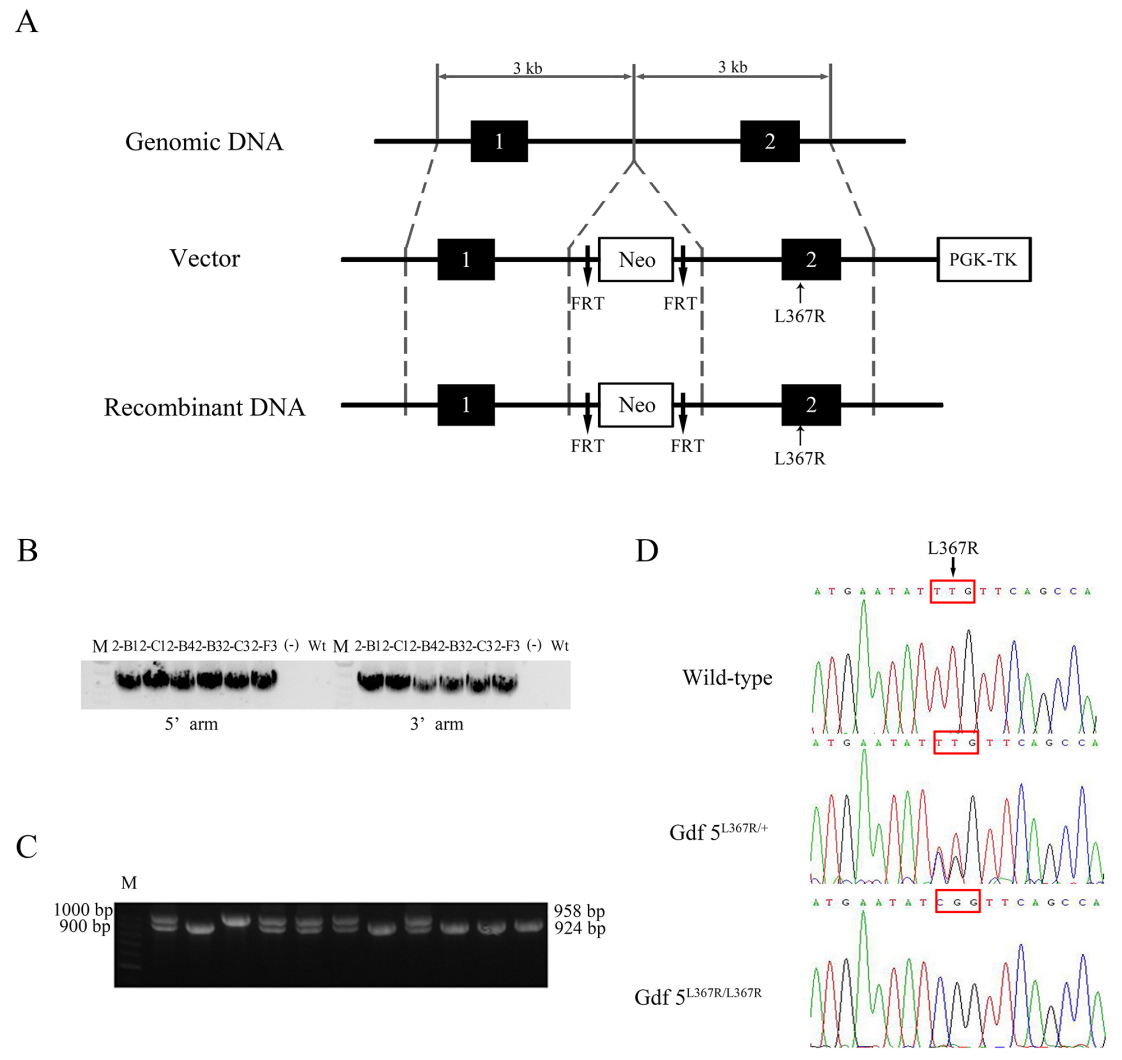
Generation of *GDF5*^*SYM1*^ mice **(A)** Strategy for the generation of *GDF5*^*L367R*^ knock-in mice. **(B)** Identification of targeted ES cell clones by PCR. **(C)** PCR amplification analysis of genomic DNA isolated from WT and Gdf5^L367R^ knock-in mice. A 958bp PCR product was present in *Gdf5*^*+/+*^ mice. A 924bp PCR product was present in *Gdf5*^*L367R/ L367R*^ knock-in mice. Both 958bp and 924bp PCR products was amplified from *Gdf5*^*L367R/+*^ mice. **(D)** The genotyping sequences of the three knock-in mice.

To generate *GDF5*^*SYM1*^ mice, we used a gene-targeting approach to replace the mouse wild type *Gdf5* with mutant *Gdf5*^*L367R*^ (the corresponding location with L373R in human) (Figure 3A). We used polymerase chain reaction (PCR) of genomic DNA to identify the targeted embryonic stem (ES) cell clones (Figure 3B). PCR and direct sequencing of genomic DNA were also used to genotype heterozygous (*Gdf5*^*L367R/+*^), homozygous (*Gdf5*^*L367R/L367R*^), and wild type *Gdf5*^*+/+*^ mice (Figure 3C, 3D).

### Skeletal abnormalities in *GDF5*^*SYM1*^ mice

**Figure 4:**
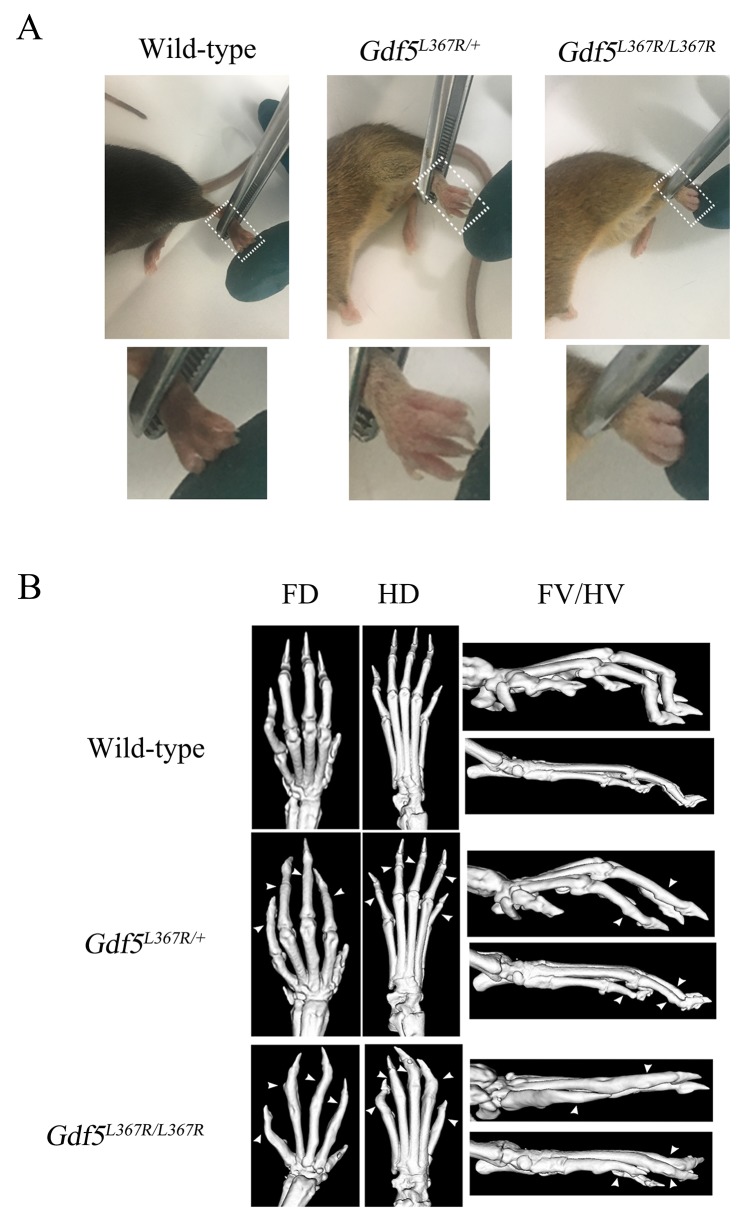
Skeletal abnormalities in *GDF5*^*SYM1*^ mice **(A)** Phenotypes of adult knock-in mice. **(B)** Micro-CT scan analysis of adult knock-in mice. (FD, Forelimbs Dorsal; HD, Hind limbs Dorsal; FV, Forelimbs Vertical; HV, Hind limbs Vertical). All the adult knock-in mice were 8 weeks old.

The features of human SYM1B patients could be clearly identified in the limbs of *GDF5*^*SYM1*^ mice by visual and radiological inspection. The length and the appearance of limbs were not showed obviously different from new born mice of three genotypes which were observed by naked eye. While after the mice were remove from milk, they began to grab food and climb on the cage. We observed that *Gdf5*^*L367R/+*^ and *Gdf5*^*L367R/L367R*^ mice would not like to climb on the cage and they often dropped from the cage because of not grabbing the cage steady. Differences became more pronounced in adult mice. *Gdf5*^*L367R/+*^ and *Gdf5*^*L367R/L367R*^ mice showed “straight hands”, with the second to fifth fingers unable to curve (Figure 4A). Since SYM1 is a dominant hereditary disease, only heterozygous mutations were found in human patients. We could see more serious damage in *Gdf5*^*L367R/L367R*^ mice, with individuals exhibiting not only straight hands, but also a fusion of the phalanges and metacarpus. When observed under Micro-CT scanning, it was clear that *Gdf5*^*L367R/+*^ mice had stiffness and adhesions between the proximal phalanx joints of the forelimbs and hindlimbs, while adhesions among the metacarpal joints, the proximal phalanx, and the distal phalanx were further observed in *Gdf5*^*L367R/L367R*^ mice (Figure 4B).

### Pronounced joint formation abnormalities in *GDF5*^*SYM1*^ mice during limb development

**Figure 5:**
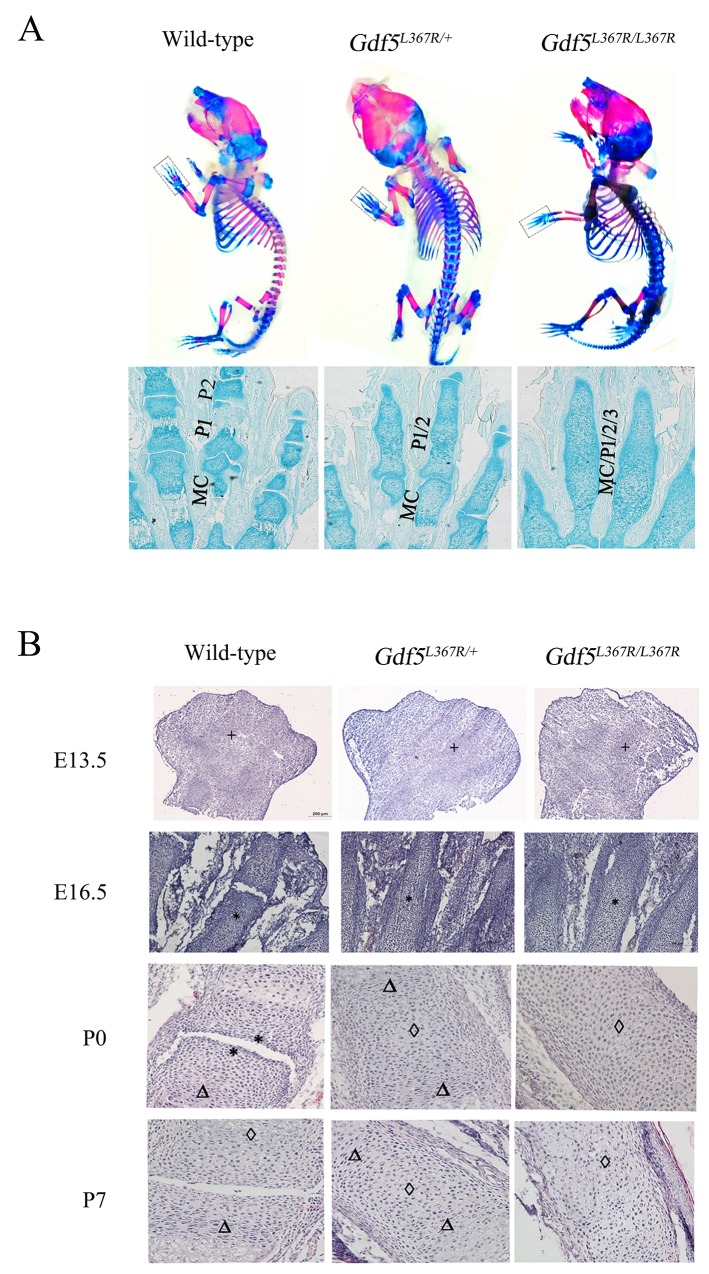
Joint formation abnormalities in *GDF5*^*SYM1*^ mice during limb development **(A)** Alcian blue-alizarin red staining on neonatal *Gdf5*^*+/+*^, *Gdf5*^*L367R/+*^, and *Gdf5*^*L367R/L367R*^ mice (P0), (MC, Metacarpals; P1, Phalanges1; P2, Phalanges2; P3, Phalanges3). **(B)** H&E staining on wild-type, heterozygous, and homozygous knock-in mice at E13.5, E16.5, P0 and P7. Chondrocyte proliferation is marked by “^*^”. “Δ” marks chondrocyte hypertrophy, and cartilage calcification is marked by “◊”.

After confirming the phenotype of SYM1, Alcian blue-alizarin red staining and histological analyses were performed to more closely examine the abnormalities in joint formation during limb development. In contrast with wild type mice, phalanges 1 and phalanges 2 were fused in *Gdf5*^*L367R/+*^ mice, and these fusions were more serious in *Gdf5*^*L367R/L367R*^ mice, including proximal phalanges (phalanges 1), middle phalanges (phalanges 2), distal phalanges (phalanges 3), and metacarpals (Figure 5A).

Furthermore, we investigated joint formation during limb development using H&E staining to observe cell morphology of the limb buds between the three genotypes on embryonic day (E) 13.5 and 16.5 and postnatal day (P) 0 and 7. It was clear that mesenchymal cells had already began to aggregate in the limb buds of wild type mice at E13.5, forming a narrow restricted surface area in the proximal interphalangeal joint by E16.5. In *Gdf5*^*L367R/+*^ and *Gdf5*^*L367R/L367R*^ mice, while mesenchymal cells still condensed at E13.5, no primary ossification centers appeared at E16.5. The interphalangeal joints continued to develop after birth in wild type mice. The restricted surface area in the proximal interphalangeal joint was clear in wild type mice at P0 but not seen in *Gdf5*^*L367R/+*^ or *Gdf5*^*L367R/L367R*^ mice. While comparing with wild type mice, chondrocyte proliferation, hypertrophy, and calcification had been seen in *Gdf5*^*L367R/+*^ and *Gdf5*^*L367R/L367R*^ mice (Figure 5B).

### Related factors alter expression during chondrosis and cartilage development

**Figure 6:**
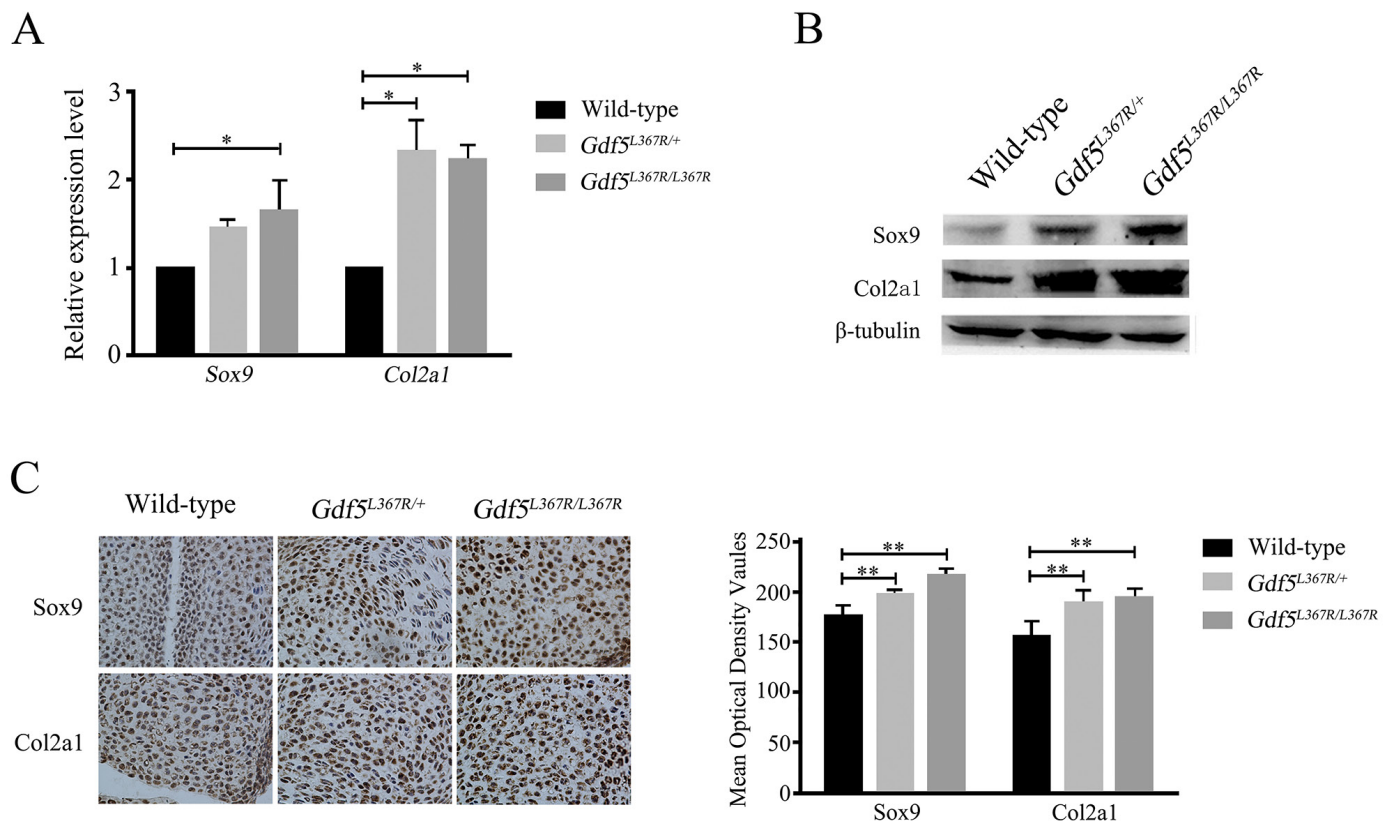
Alterations of the expression of related factors Sox9 and Col2a1 in *GDF5*^*SYM1*^ knock-in mice **(A)** Real time qPCR analysis of Sox9 and Col2a1 expression in *GDF5*^*SYM1*^ mice. **(B)** Western blot analysis of Sox9 and Col2a1 expression in *GDF5*^*SYM1*^mice. **(C)** Histochemistry analysis of Sox9 and Col2a1 expression in *GDF5*^*SYM1*^ mice and their respective mean optical density analysis. (*P <0.05, ** *P* <0.01).

We detected related factors during chondrosis and cartilage development in wild type, *Gdf5*^*L367R/+*^, and *Gdf5*^*L367R/L367R*^ mice. Real-time RT-PCR, western blot, and histochemistry all showed a higher expression of Sox9 and Col2a1 in wild type mice than in *Gdf5*^*L367R/+*^ and *Gdf5*^*L367R/L367R*^ mice (Figure 6). This result was in accordance with our morphologic observations, and suggests that the mutant chondrocytes proliferate continuously instead of differentiating and forming ossification centers.

## DISCUSSION

In the above project, we describe a SYM1 mouse model (*GDF5*^*SYM1*^) which carries the *Gdf5*^*L367R*^ mutation, the corresponding mutation to the human *GDF5*^*L373R*^ mutation found in a SYM1 family previously reported on by our group. GDF5 is best known for its role in early chondrogenesis and joint formation. It has been shown to regulate the proliferation and differentiation of chondrogenic tissue during bone and cartilage development, and it is also associated with osteoarthritis [17]. The role of GDF5 in the embryonic development of the phalanges of tetrapods is very important [3, 18, 19]. GDF5 stimulates the expression of chondrogenic markers at embryonic day 14 [20]. GDF5 is expressed by interzone cells in highly dynamic spatiotemporal patterns, and the joints form through continuous influx of Gdf5-positive cells following an influx model [21]. Both *in vitro* and *in vivo* studies have shown that GDF5 induces chondrogenesis. One study showed that cultured embryonic chick mesenchymal cells overexpressing GDF5 distinctly presented mature hypertrophic chondrocytes [22]. Previous studies have also shown that GDF5 could stimulate the expression of cartilage anabolic genes [23]. In our study, the expression of chondrogenic markers COL2A1 and SOX9 were distinctly higher in cells infected by GDF5^L373R^ than wild type GDF5. The corresponding increase in the phosphorylation of SMAD1/5/8 in cells infected by GDF5^L373R^ suggested that the GDF5^L373R^ mutation may affect the interaction between GDF5 and bone morphogenetic protein receptors. Mature GDF5 binding to BMPR1B would induce downstream signaling cascades and SMAD-dependent signal transduction to stimulate chondrocyte proliferation [24]. The higher expression of Sox9 and Col2a1 were also observed in *Gdf5*^*L367R/+*^ and *Gdf5*^*L367R/L367R*^ mice. Meanwhile *Gdf5*^*L367R/+*^ and *Gdf5*^*L367R/L367R*^ mice showed chondrocyte proliferation, hypertrophy and calcification. We also noticed that, comparing with wild type, in *Gdf5*^*L367R/+*^ and *Gdf5*^*L367R/L367R*^ mice the cells in Proximal phalanx showed endoplasmic reticulum dilatation (data was not showed). This reminded us that the abnormal unfolded mutant *Gdf5* might activate endoplasmic reticulum stress which is closely interconnected with autophagy and apoptosis. According to report in recent years, autophagy could regulate the secretion of Col2a1 [25]. It might also be part of reason for the increase of Col2a1 in *Gdf5*^*L367R/+*^ and *Gdf5*^*L367R/L367R*^ mice. All the results suggested that the gain-of-function mutation GDF5^L373R^ could cause the persistence of cartilage.

GDF5 mutations have previously been associated with several kinds of inherited skeletal diseases. Some of them are autosomal recessive disorders, such as Acromesomelic dysplasia Grebe type, Acromesomelic dysplasia Hunter-Thompson type, and Du Pan syndrome [26, 27], whereas others are autosomal dominant disorders, including Proximal symphalangism, Symphalangism proximal 1B, Brachydactyly type A2, Brachydactyly type C, and Multiple synostoses syndrome 2 [5, 28]. Brachydactyly type A1 is also associated with GDF5 mutations, which could be autosomal dominant or recessive. All these GDF5-related diseases can be classified into two groups according to the functional change of GDF5. Most mutations result in the deactivation of GDF5, causing the abnormal skeletal development seen in disorders such as Brachydactyly type A2. Alternately a gain-of-function mutation in GDF5 causes the persistence of cartilage and results in Proximal symphalangism or Multiple synostoses syndrome.

These different phenomena caused by GDF5 mutations can be associated with the position in which the mutations exists on GDF5. Similar to other BMPs, GDF5 is composed of 501 amino acids (aa), including an N terminal signal peptide domain (1-27 aa), a propeptide region, a putative polybasic processing site (28-381 aa) and a 120 amino acid polypeptide at the carboxyl terminus (382-501 aa). The GDF5 pro-form undergoes endopeptidase cleavage at the polybasic processing site (RRKKRR). Upon processing, the remaining 120 amino acids form the mature GDF5 protein [29], which then dimerizes to bind with receptors [30, 31]. With 354 residues composing the proregion, GDF5 belongs to the group of growth factors with large proregions. Although part of the proregion of GDF5 is cleaved before the mature GDF5 protein is biologically active, the importance of the region becomes apparent when mutations in proregion of GDF5 were reported. GDF5 mutation R380Q can lead to brachydactyly type A2; M173V/L176P/S204R/R301stop can all lead to brachydactyly type C; R378Q can lead to Du Pan syndrome [10, 32–34]. Tino Thieme speculated that the core domain of GDF5 encompasses residues 194-347 of the proregion. Mutations in this core subdomain might lead to reduced thermodynamic stability [6]. However, the mutation investigated in this study, GDF5-L373R, is not in the core subdomain. We found that this mutation results in increased proteolysis efficiency and upregulation of SMAD signal transduction in ATDC5 cells. We used methods of Western blot and histochemistry to detect the protein level of mature Gdf5 in *GDF5*^*SYM1*^ mice at E13.5 as well. Unfortunately, the increase of mature Gdf5 in *GDF5*^*SYM1*^ mice were not observed (data was not showed). Except for the limited quantity of Gdf5 extracted from limb buds of the fetal mice, the most possible reason for the result might be the time points. Although the time point of joint development is E11.5-E13.5, Gdf5 plays its role much earlier than that. So more accurate monitoring need to be done during the early embryo development on the *GDF5*^*SYM1*^ mice.

The mouse is a convenient animal model for studying human diseases. The mouse and human GDF5 precursor proteins share 91% identity, and the mature proteins differ by only 1 amino acid. Although GDF5-deficient mice have been identified since 1994 [35] and brachypodism and ankyloses were observed in mutant mice with a W408R substitution in the highly conserved region of the active signaling domain of the Gdf5 protein [36], no mouse model of SYM1 has been previously made. Until now, five GDF5 mutations have been reported in humans which result in SYM1, and L373R is the only mutation located within the GDF5 proregion. The *Gdf5*^*L367R*^ knock-in mouse model completely replicates the human SYM1 phenotypes. *Gdf5*^*L367R/+*^ mice show inflexibility in their fingers and toes, similar to SYM1 patients. The results of Micro-CT scanning, Alcian blue-alizarin red staining, and histological analysis identified that the ankylosis was caused by abnormal joint fusions. After observing their skeletal development, we found that *Gdf5*^*L367R/+*^ mice failed to develop primary ossification centers even at E16.5, though the mesenchymal cells properly condensed at E13.5. The interphalangeal joints present high chondrocyte proliferation, hypertrophy, and calcification after birth, with high expression of chondrogenic markers. The *GDF5*^*SYM1*^ mouse model also provides us with viable *Gdf5*^*L367R/L367R*^ mice to analyze. Homozygous phenotypes are limited in humans due to the mode of inheritance of SYM1. In these mice, the metacarpal joints, the proximal phalanx, and the distal phalanx are fused together, similar to the multiple synostoses syndrome (SYNS).

While we did not examine the hearing of the mice to see whether they experience the progressive conductive deafness seen in some patients, we did find that both *Gdf5*^*L367R/+*^ and *Gdf5*^*L367R/L367R*^ mice were less alert to their environment than wild type mice. It is possible that some kind of conductive hearing loss occurs in the *GDF5*^*SYM1*^ mice, which makes the hearing in *Gdf5*^*L367R/+*^ and *Gdf5*^*L367R/L367R*^ mice less sensitive than the wild type mice and results in an observable loss of alertness.

## MATERIALS AND METHODS

### Cell culture, cloning, and lentivirus transduction

ATDC5 chondrogenic cells were cultured in DMEM/F12 medium (1:1 mixture of DMEM and Ham’s F-12 medium), with 5% fetal bovine serum (Gibco, ThermoFisher Scientific, Germany).

The coding sequences of both wild type and mutant *GDF5*, both lacking the signal peptide, were amplified by PCR and cloned into pcDNA3.1 vectors, making pcDNA3.1-GDF5-WT^delSP^ and pcDNA3.1-GDF5-MT^delSP^ respectively. After confirming the sequencing, all plasmids were transfected into ATDC5 cells using Lipofectamine 2000 (Invitrogen, CA, USA). The transfected cells were cultured and processed for Western blot assays.

Lentiviruses containing wild type GDF5 (GDF5-WT), mutant GDF5 (GDF5-L373R), or negative control (NC) were produced by Genechem (China). ATDC5 cells were seeded in 24-well culture plates at a density of 2×10^5^ cells/well and incubated in 500 μl medium at 37°C. All lentivirus constructs were used at 20 MOI (multiplicity of infection) to infect ATDC5 cells. The original medium was replaced with DMEM/F12 medium after culturing for 1 day. The transfected cells were cultured and processed for the following Western blot assays.

### Real-time RT-PCR analyses

Total RNA was isolated from tissues using Trizol Reagent (Invitrogen, CA). The reverse transcription (RT) step was performed with a PrimeScript™ II Reverse Transcriptase kit (TaKaRa, China).

Real-time RT-PCR was performed with a total volume of 25 μl, containing 12.5 μl SYBR Premix Ex Taq (TaKaRa, China), 50 ng cDNA (5 μl), and 4 x 2.5 μl primers (250 nM each) in each tube. Primer information can be found in Table 1. The reaction conditions were 95°C for 20 s, followed by 40 cycles of amplification at 95°C for 30 s, 95°C for 5 s, and 60°C for 20 s, and the reactions were carried out by the Rotor-Gene 2000 Real-Time Cycler (Applied Biosystems, USA).

**Table 1:** Primers used in real-time RT-PCR analysis

Gene	Primer sequence
*Sox9*	Forward 5’-ACTTGCACAACGCCGAGC-3’ Reverse 5’-CGCGGCTGGTACTTGTAATCC-3’
*Col2a1*	Forward 5’- TGGACGATCAGGCGAAACC -3’ Reverse 5’- GCCAGCAAAGGCGGACAT -3’
*Gapdh*	Forward 5’- CATTGTGGAAGGGCTCATGAC -3’ Reverse 5’- GGATGCAGGGATGATGTTCTG -3’

All samples were run in triplicate in separate tubes. Relative expression levels were calculated using the comparative threshold cycle (CT) method, with glyceraldehyde-3-phosphate dehydrogenase (Gapdh) used as a house-keeping gene.

### Western blot analyses

Transfected cells or limbs of knock-in mice were lysed in ice-cold radio immunoprecipitation assay (RIPA) buffer containing 0.01% protease (Sigma-Aldrich, USA) for 30 min at 4°C. Cell lysis was centrifuged at 12000 rpm for 15 min at 4°C and the protein concentrations were quantified using the Bicinchoninic Acid (BCA) kit (Sigma-Aldrich, USA). SDS-PAGE and western blotting were performed using standard techniques. The primary antibodies of GDF5, SOX9, COL2A1, SMURF1, ID1 and phosphorylated SMAD1/5/8 (Santa Cruz, USA) were used at a dilution of 1:1000, and β-tubulin (Abcam, USA) was used at a dilution of 1:10000 in PBS-Tween 20.

### Duolink in situ interaction assay

Interactions between GDF5 and BMPR1B were detected using the Duolink^®^ In Situ – Fluorescence kit (Sigma-Aldrich) after transduction with lentivirus NC, GDF5-WT, or GDF5-L373, following the instructions of the manufacturer. Briefly, cells were incubated with primary antibodies against GDF5 and BMPR1B (1:100 dilution; Santa Cruz, Biotechnology), then washed with wash buffer for 2×5 min. Two PLA probes (1:5 dilution) were applied and incubated for 60 min at 37°C. Ligase was diluted at 1:40 in the solution and incubated for 30 min at 37°C. The samples were then incubated for 100min with Polymerase at 37°C and mounted with Duolink In Situ Mounting Medium with DAPI for 15 min. The cells were visualized using a confocal microscope system.

### Generation of *Gdf5*^*L367R*^ knock-in mice

The L367R mutation was introduced into exon 2 using site-directed mutagenesis. The targeting vector plasmid pBR322 for the Gdf5 knock-in mice also contains a 3 kb 5’homologous arm, a Pgk-neomycin (neo) cassette flanked with two FRT sites between exon1 and exon2, a 3 kb 3’homologous arm, and an MC1-thymidine kinase cassette outside of the homology region. The homologous arms were obtained from the 129S6 Mouse BAC plasmid by homologous recombination and ampicillin screening. ES cells were electroporated with 35μg linearized targeting vector and selected in medium containing G418 (300mg/L) and ganciclovir (2 μmol/L) 24 h and 48 h after electroporation, respectively. Correctly targeted ES cell clones were screened by gDNA extraction and PCR genotyping. ES cell clones that showed the desired chromosomal integration were injected into blastocysts, which were then transplanted into pseudo pregnant female mice. Chimeric males were mated with C57BL/6 females and the resulting 5 offspring were genotyped. The Frt-flanked Neo cassette was removed by crossed with Flp mice, produced by Shanghai Nanfang Model Biology.

### Skeletal analysis

Skeletons of newborn mice were fixed in 4% paraformaldehyde/PBS overnight at 4°C, dehydrated, then stained with Alizarin red/Alcian blue staining. For Safranin fast green staining histology, adult mouse limbs were fixed and dehydrated, then 6 μm sections were mounted on glass slides, rehydrated, and stained for 12 min separately (10% Safranin O, 0.5% fast green). Living animal micro-CT imaging was performed setting micro-CT slices at 40 μm that were then reconstructed in 3D arrays.

### Histology and immunohistochemistry determination

All animals were euthanized according to protocols approved by the Chinese Institutional Animal Care and Use Committee (CIACUC). Healthy newborn mice from the same brood with three genotypes were used in the research. Intact or dissected embryo limbs or skinned and decalcified newborns were sectioned using a frozen slicer. For general morphology, sections were stained with hematoxylin and eosin. After blocking with 5% normal goat serum, sections were incubated with anti-sox9 (1:200; Santa, USA) or anti-col2a1 (1:300; Abcam, USA) overnight at 4°C. Sections were incubated for 30 min with secondary antibody followed by amplification with SABC (Boster, China), and visualization with DAB (Boster, China).

### Statistical analysis

All data presented are representative of at least three independent experiments for each condition. Statistical analyses were performed using Student’s *t*-test. P values of less than 0.05 were considered statistically significant.

## References

[1] Plett SK, Berdon WE, Cowles RA, Oklu R, Campbell JB (2008). Cushing proximal symphalangism and the NOG and GDF5 genes. Pediatr Radiol.

[2] Elkington SG, Huntsman RG (1967). The Talbot fingers: a study in symphalangism. Br Med J.

[3] Jin L, Li X (2013). Growth differentiation factor 5 regulation in bone regeneration. Curr Pharm Des.

[4] Feng C, Liu H, Yang Y, Huang B, Zhou Y (2015). Growth and differentiation factor-5 contributes to the structural and functional maintenance of the intervertebral disc. Cell Physiol Biochem.

[5] Stange K, Thieme T, Hertel K, Kuhfahl S, Janecke AR, Piza-Katzer H, Penttinen M, Hietala M, Dathe K, Mundlos S, Schwarz E, Seemann P (2014). Molecular analysis of two novel missense mutations in the GDF5 proregion that reduce protein activity and are associated with brachydactyly type C. J Mol Biol.

[6] Thieme T, Patzschke R, Job F, Liebold J, Seemann P, Lilie H, Balbach J, Schwarz E (2014). Biophysical and structural characterization of a folded core domain within the proregion of growth and differentiation factor-5. FEBS J.

[7] Schwaerzer GK, Hiepen C, Schrewe H, Nickel J, Ploeger F, Sebald W, Mueller T, Knaus P (2012). New insights into the molecular mechanism of multiple synostoses syndrome (SYNS): mutation within the GDF5 knuckle epitope causes noggin-resistance. J Bone Miner Res.

[8] Seo SH, Park MJ, Kim SH, Kim OH, Park S, Cho SI, Park SS, Seong MW (2013). Identification of a GDF5 mutation in a Korean patient with brachydactyly type C without foot involvement. Ann Lab Med.

[9] Umair M, Rafique A, Ullah A, Ahmad F, Ali RH, Nasir A, Ansar M, Ahmad W (2017). Novel homozygous sequence variants in the GDF5 gene underlie acromesomelic dysplasia type-grebe in consanguineous families. Congenit Anom (Kyoto).

[10] Douzgou S, Lehmann K, Mingarelli R, Mundlos S, Dallapiccola B (2008). Compound heterozygosity for GDF5 in Du Pan type chondrodysplasia. Am J Med Genet A.

[11] Yang W, Cao L, Liu W, Jiang L, Sun M, Zhang D, Wang S, Lo WH, Luo Y, Zhang X (2008). Novel point mutations in GDF5 associated with two distinct limb malformations in Chinese: brachydactyly type C and proximal symphalangism. J Hum Genet.

[12] Leonidou A, Irving M, Holden S, Katchburian M (2016). Recurrent missense mutation of GDF5 (p.R438L) causes proximal symphalangism in a British family. World J Orthop.

[13] Seemann P, Brehm A, Konig J, Reissner C, Stricker S, Kuss P, Haupt J, Renninger S, Nickel J, Sebald W, Groppe JC, Ploger F, Pohl J (2009). Mutations in GDF5 reveal a key residue mediating BMP inhibition by NOGGIN. PLoS Genet.

[14] Wang X, Xiao F, Yang Q, Liang B, Tang Z, Jiang L, Zhu Q, Chang W, Jiang J, Jiang C, Ren X, Liu JY, Wang QK (2006). A novel mutation in GDF5 causes autosomal dominant symphalangism in two Chinese families. Am J Med Genet A.

[15] Seemann P, Schwappacher R, Kjaer KW, Krakow D, Lehmann K, Dawson K, Stricker S, Pohl J, Ploger F, Staub E, Nickel J, Sebald W, Knaus P (2005). Activating and deactivating mutations in the receptor interaction site of GDF5 cause symphalangism or brachydactyly type A2. J Clin Invest.

[16] Wu Q, Chen D, Zuscik MJ, O'Keefe RJ, Rosier RN (2008). Overexpression of Smurf2 stimulates endochondral ossification through upregulation of beta-catenin. J Bone Miner Res.

[17] Zhang R, Yao J, Xu P, Ji B, Luck JV, Chin B, Lu S, Kelsoe JR, Ma J (2015). A comprehensive meta-analysis of association between genetic variants of GDF5 and osteoarthritis of the knee, hip and hand. Inflamm Res.

[18] Storm EE, Kingsley DM (1999). GDF5 coordinates bone and joint formation during digit development. Dev Biol.

[19] Wolfman NM, Hattersley G, Cox K, Celeste AJ, Nelson R, Yamaji N, Dube JL, DiBlasio-Smith E, Nove J, Song JJ, Wozney JM, Rosen V (1997). Ectopic induction of tendon and ligament in rats by growth and differentiation factors 5, 6, and 7, members of the TGF-beta gene family. J Clin Invest.

[20] Yin S, Cen L, Wang C, Zhao G, Sun J, Liu W, Cao Y, Cui L (2010). Chondrogenic transdifferentiation of human dermal fibroblasts stimulated with cartilage-derived morphogenetic protein 1. Tissue Eng Part A.

[21] Shwartz Y, Viukov S, Krief S, Zelzer E (2016). Joint development involves a continuous influx of Gdf5-positive cells. Cell Rep.

[22] Coleman CM, Tuan RS (2003). Growth/differentiation factor 5 enhances chondrocyte maturation. Dev Dyn.

[23] Enochson L, Stenberg J, Brittberg M, Lindahl A (2014). GDF5 reduces MMP13 expression in human chondrocytes via DKK1 mediated canonical Wnt signaling inhibition. Osteoarthritis Cartilage.

[24] Miyazono K, Kamiya Y, Morikawa M (2010). Bone morphogenetic protein receptors and signal transduction. J Biochem.

[25] Cinque L, Forrester A, Settembre C (2016). Autophagy gets to the bone. Cell Cycle.

[26] Faiyaz-Ul-Haque M, Ahmad W, Zaidi SH, Haque S, Teebi AS, Ahmad M, Cohn DH, Tsui LC (2002). Mutation in the cartilage-derived morphogenetic protein-1 (CDMP1) gene in a kindred affected with fibular hypoplasia and complex brachydactyly (DuPan syndrome). Clin Genet.

[27] Martinez-Garcia M, Garcia-Canto E, Fenollar-Cortes M, Aytes AP, Trujillo-Tiebas MJ (2015). Characterization of an acromesomelic dysplasia, Grebe type case: novel mutation affecting the recognition motif at the processing site of GDF5. J Bone Miner Metab.

[28] Degenkolbe E, Konig J, Zimmer J, Walther M, Reissner C, Nickel J, Ploger F, Raspopovic J, Sharpe J, Dathe K, Hecht JT, Mundlos S, Doelken SC (2013). A GDF5 point mutation strikes twice--causing BDA1 and SYNS2. PLoS Genet.

[29] Chang SC, Hoang B, Thomas JT, Vukicevic S, Luyten FP, Ryba NJ, Kozak CA, Reddi AH, Moos M (1994). Cartilage-derived morphogenetic proteins. New members of the transforming growth factor-beta superfamily predominantly expressed in long bones during human embryonic development. J Biol Chem.

[30] Hauburger A, von Einem S, Schwaerzer GK, Buttstedt A, Zebisch M, Schraml M, Hortschansky P, Knaus P, Schwarz E (2009). The pro-form of BMP-2 interferes with BMP-2 signalling by competing with BMP-2 for IA receptor binding. FEBS J.

[31] Erlacher L, McCartney J, Piek E, ten Dijke P, Yanagishita M, Oppermann H, Luyten FP (1998). Cartilage-derived morphogenetic proteins and osteogenic protein-1 differentially regulate osteogenesis. J Bone Miner Res.

[32] Ploger F, Seemann P, Schmidt-von Kegler M, Lehmann K, Seidel J, Kjaer KW, Pohl J, Mundlos S (2008). Brachydactyly type A2 associated with a defect in proGDF5 processing. Hum Mol Genet.

[33] Schwabe GC, Turkmen S, Leschik G, Palanduz S, Stover B, Goecke TO, Mundlos S (2004). Brachydactyly type C caused by a homozygous missense mutation in the prodomain of CDMP1. Am J Med Genet A.

[34] Farooq M, Nakai H, Fujimoto A, Fujikawa H, Kjaer KW, Baig SM, Shimomura Y (2013). Characterization of a novel missense mutation in the prodomain of GDF5, which underlies brachydactyly type C and mild Grebe type chondrodysplasia in a large Pakistani family. Hum Genet.

[35] Storm EE, Huynh TV, Copeland NG, Jenkins NA, Kingsley DM, Lee SJ (1994). Limb alterations in brachypodism mice due to mutations in a new member of the TGF beta-superfamily. Nature.

[36] Masuya H, Nishida K, Furuichi T, Toki H, Nishimura G, Kawabata H, Yokoyama H, Yoshida A, Tominaga S, Nagano J, Shimizu A, Wakana S, Gondo Y (2007). A novel dominant-negative mutation in Gdf5 generated by ENU mutagenesis impairs joint formation and causes osteoarthritis in mice. Hum Mol Genet.

